# The MSC–RSV/HMPV interface: impacts on pulmonary homeostasis, remodeling, and risk of non-atopic asthma and COPD

**DOI:** 10.3389/fcimb.2026.1952735

**Published:** 2026-10-30

**Authors:** Rocio Tirado Mendoza

**Affiliations:** Laboratory of Viral Pathogens and Stem Cells, Faculty of Medicine, Department Microbiology and Parasitology, National Autonomous University of Mexico (UNAM), Mexico City, Mexico

**Keywords:** chronic obstructive pulmonary disease, human metapneumovirus (HMPV), mesenchymal stem cells, non-atopic asthma, respiratory syncytial virus, respiratory tract infections, neutrophil extracellular traps

## Abstract

Respiratory tract infections continue to represent a major cause of pediatric morbidity and mortality. Respiratory Syncytial Virus (RSV) and Human Metapneumovirus (HMPV) are highlighted as key etiologic agents of bronchiolitis and pneumonia, particularly in children under two years old. Recurrent infections during the second year of life may be associated with significant histopathological damage and might also be a risk factor for developing chronic pulmonary diseases, such as non-atopic asthma. In adults, these viruses similarly elevate clinical burden on the elderly and individuals with Chronic Obstructive Pulmonary Disease (COPD), causing the exacerbation of respiratory signals and symptoms and, consequently, promoting a chronic inflammatory response that modifies the lung tissue and disease progression. Tissue repair and the homeostasis recovery might be associated with the participation of Mesenchymal Stem Cells (MSCs), both lung resident and migratory MSCs, these cells are characterized by their plasticity and immunomodulatory capacity, which are relevant to restoring tissue homeostasis after injury. However, RSV and HMPV may also infect MSCs, raising the question that infected MSCs could lose immunomodulatory efficacy and plasticity, thereby affecting tissue renewal. This review aims to provide an overview of the effect of respiratory viral infections, their implications in the lung tissue damage and the possible participation of MSCs in the recovery of lung homeostasis by tissue repair and immune response modulation.

## 1 Introduction

In Mexico, according to the Conventional Epidemiological Surveillance System and through the epidemiological bulletin (week 40/2025 to week 20/2026) (Secretaría de Salud, 2025), health authorities report 17,181,856 new cases of Acute Respiratory Infections (ARIs), of which 205,938 were confirmed to be caused by viral etiology. Human respiratory viruses such as Respiratory Syncytial Virus (RSV) and Human Metapneumovirus (HMPV) are the most frequent etiological agents of acute respiratory infections, both affecting mainly children under two years old (Jobe et al., 2025; World Health Organization, 2025a), and causing respiratory infections that range from asymptomatic to severe cases (World Health Organization, 2025a, 2025b, 2025c, 2025d).

Moreover, both viruses are associated with reinfections that could cause moderate to severe infections (World Health Organization, 2025c; World Health Organization, 2025d); these last ones produce lung injury which might be associated to the development of chronic diseases such as non-atopic asthma (Mikhai and Grayson, 2019; Manti and Piedimonte, 2022). Also, both viruses are considered risk factors that exacerbate the signs and symptoms of chronic pulmonary diseases like Chronic Obstructive Pulmonary Disease, which mainly affects adults (Rudd et al., 2017; Penders et al., 2025). These chronic diseases are associated with the inflammatory process, which could be mediated by soluble factors, immune cells and the production of NETs (Varricchi et al., 2022; Katsoulis et al., 2024), all of which induce lung damage, affecting the tissue homeostasis. For the reestablishment of the lung homeostasis, It should be noted that the participation of the mesenchymal stem cells. First, they participate in the modulation of the immune response. Secondly, they are multipotential cells, meaning they have the capacity to differentiate into multiple lineages; and therefore, stimulate tissue repair by promoting homeostasis and facilitating tissue recovery (Sveiven and Nordgren, 2020; Walewska et al., 2025).

## 2 Respiratory virus infections as triggers of physiopathology during non-atopic asthma and COPD

Among the leading causes of morbidity and mortality from infectious diseases worldwide are ARIs, which are considered the main reason for medical consultation and hospitalization, especially in pediatrics (Chena et al., 2024). The PAHO notes that approximately four million people die annually from these infections (Chena et al., 2024; Pan Amerian Health Organization, 2025), with children aged 0 to 2 years old and older adults being the most susceptible to this type of infection (Chena et al., 2024; Pan Amerian Health Organization, 2025). Mortality from ARIs is due to severe clinical complications such as pneumonia, bronchitis, and bronchiolitis. Pneumonia is the leading cause of childhood mortality worldwide in children under 5 years of age (World Health Organization, 2025c). ARIs are caused by various etiological agents, including viruses that cause diseases ranging from asymptomatic to severe. Pediatric patients with severe respiratory infections exhibit histopathological damage, which predisposes them to the development of chronic pulmonary conditions such as non-atopic asthma (Turner et al., 2024; Rosas-Salazar et al., 2023). Likewise, ARIs are a risk factor that contributes to exacerbating the signs and symptoms of other conditions such as bronchopulmonary dysplasia and chronic obstructive pulmonary disease (COPD) (Johnston et al., 1995; Sigurs et al., 2010). COPD is the fourth leading cause of morbidity and mortality in the world, and it is projected that by 2030 it will become the third leading cause of death due to continuous exposure to risk factors and population aging (Luna et al., 2020). In Mexico, there are 3.2 million people with COPD; it is the 9th leading cause of death in the general population and the 6th leading cause of death in women. Approximately 20,000 deaths per year are reported, with a cost of 347 million pesos in hospitalizations, treatment, and loss of work productivity due to absences. Recurrent respiratory infections induce a recurring inflammatory process that leads to anatomical remodeling. These structural changes involve histological alterations, including increased airway smooth muscle, thickening of the subepithelial reticular lamina, fibrosis, angiogenesis, neural proliferation, and mucosal metaplasia (Cervantes et al., 2018).

Some reports demonstrate that severe infection by respiratory syncytial virus (RSV) during childhood are associated with metabolic changes in the respiratory epithelium, as well as alterations and dysfunction of the epithelial barrier, which may affect lung development (Rosas-Salazar et al., 2023; Vandini et al., 2017; Solís-Rodríguez et al., 2020; Kuang et al., 2024). The combination of factors such as changes in the respiratory microbiome, alterations in the respiratory epithelial barrier, and a deficient immune response (Th1 and Th17) caused by RSV infection predisposes children under two years of age to allergen sensitization, an exaggerated response to environmental pollutants, and recurrent respiratory infections all of which are risk factors for the development of non-atopic asthma (Hammad et al., 2009). Moreover, severe RSV infection may promote the differentiation of Th17 lymphocytes, which may be associated with its pathophysiology and its resultant sequelae such as airway and hyper-reactivity (AHR) (Qin et al., 2019). Also, these cells could contribute to inflammatory disorders that afflict the respiratory tract, such as asthma and chronic obstructive pulmonary disease (COPD). Additionally, it should be noted that the increased production of the Th17-related cytokines, such as IL-17A, IL-22 and IL-23 in COPD patients, reflects the involvement of Th17 lymphocytes in initiating and driving the disease process. The excess of IL-17 is not exclusive to these patients, there are some reports that point out, both in animal models and in human patients, that the overproduction of IL-17 by Th17 might be associated with neutrophil dominated asthma and with cortisone-resistant severe airway and AHR (Bystrom et al., 2013). Some reports from animal models have shown that a lack of, or inadequate, responses by the Th1 subset of T lymphocytes enhances Th17-mediated responses and that this is detrimental during RSV co-infection in experimental asthma (Bystrom et al., 2013). Finally, RSV infection may increase the risk of asthma morbidity, but the underlying mechanism remains unknown. In allergic asthma, a critical event is the activation of Th cells, leading to a predominance of Th2 cytokines over Th1 cytokines. A recent study suggests that Th1, as well as Th2 cytokines, may promote asthma (Qin et al., 2011). Equally important, severe RSV infections promote the overexpression and sensitization of TLR-4, which cause an exaggerated immune response with bronchial hyperreactivity upon reinfection with this virus (Gern, 2004). What’s more, genetic susceptibility has been described, particularly polymorphisms in cytokines or receptors, which predispose individuals to the development of asthma following an RSV infection (Cervantes et al., 2018).

Likewise, the HMPV also has a relevant role as the second human respiratory virus, only after RSV infection, especially in pediatric clinical practice and in vulnerable populations, as well as for its frequency and clinical importance. It is common for HMPV infection to appear repeatedly throughout life; however, occasionally it could complicate, causing severe bronchiolitis and pneumonia, requiring hospitalization, intensive care unit (ICU) admission, and intubation (Rudd et al., 2017; Williams et al., 2004; Kusel et al., 2007). Moreover, these respiratory complications damage lung tissue, thus it may play a role in initiating asthma development or recurrent wheezing in children (Rudd et al., 2017). Evidence suggests that HMPV infection during infancy may be a significant risk factor for the development of preschool asthma within the first 5 years of life, like what has been reported for RV and RSV (Sigurs et al., 2000; Wu and Hartert, 2011; Kan-o et al., 2017). Nevertheless, these complications are not exclusive to children, adults can also experience respiratory compromise/difficulties. These severe respiratory manifestations may be particularly exacerbated in adults with chronic obstructive pulmonary disease (COPD) and in children with asthma, as well as people with a weakened immune system (Sigurs et al., 2000; Wu and Hartert, 2011; Kan-o et al., 2017). Finally, some reports point out that HMPV was detected in 18.2% of hospitalized COPD patients and the authors demonstrated that rate of detection (Hamelin et al., 2006).

In the case of HMPV infection, animal models have been highly useful, particularly the murine model, because these animals are susceptible to HMPV infection. This model has helped explain how HMPV may cause long-term lung damage (Rudd et al., 2017; Soto et al., 2018). The authors reported that HMPV infection could induce airway remodeling and mucus production, suggesting that HMPV may contribute to the development of more severe lung disease (Rudd et al., 2017). Other researchers have also reported asthma-like manifestations, including airway obstruction and airway hyperresponsiveness. These respiratory manifestations are associated with chronic inflammatory processes that may persist for months (Liu et al., 2009; Herd et al., 2006¸ Bugatti et al., 2020). Furthermore, the authors reported the presence of viral RNA, which could be considered a trigger contributing to the chronic inflammatory process and, consequently, to lung damage (Liu et al., 2009). In addition, some authors have described how primary HMPV infection may alter the response to pharmacological treatment for exacerbated acute hyperresponsiveness (AHR), suggesting that HMPV may be responsible for this type of pathological change long after the primary infection (Rudd et al., 2017; Liu et al., 2009; Herd et al., 2006). This, in turn, could help explain the subsequent development of asthma in children following hospitalization for HMPV infection. Previously, we described a role for HMPV as a trigger of asthma and highlighted the relevance of viral RNA persistence in chronic lung disease. The next question is how and where HMPV persists. Some authors have suggested that viral persistence within neuronal projections in the lung may explain the prolonged presence of HMPV (Herd et al., 2006; González et al., 2017**;** Bugatti et al., 2020). Thus, the virus may establish persistence in an immunoprivileged site that favors its long-term presence. In addition to persisting in these sites, HMPV modulates the immune response, suggesting that it contributes to the establishment of persistent infection. HMPV infection alters the innate immune response and may also induce an aberrant adaptive immune response. Several reports have shown that HMPV infection induces a Th1-type response during the early phase of respiratory infection; later, the host mounts a Th2-type response characterized by high levels of interleukin-10 (IL-10), low levels of IFN-γ, and a delayed cytotoxic T-cell response (Alvarez and Tripp, 2005; Global Initiative for Asthma, 2026; González et al., 2017). Finally, at least two factors may play a key role in the development of non-atopic asthma and the exacerbation of AHR in both asthma patients and COPD patients.

## 3 Non atopic asthma might be a consequence of human respiratory syncytial virus and/or human metapneumovirus infection?

Asthma is a chronic respiratory disease that affects multiple age groups and has become increasingly prevalent. It is a multifactorial disorder and remains one of the leading causes of outpatient and emergency care visits. Globally, asthma affects more than 260 million people, representing approximately 5% to 8% of the population, and accounts for more than 450,000 deaths each year (García González et al., 2025). In Mexico, according to the Instituto Nacional de Enfermedades Respiratorias (INER), asthma affects an estimated 8.5 million people, with a prevalence ranging from 5% to 12%. For the 2020–2024 period, asthma-related deaths showed a downward trend, with 188 deaths documented in the most recent report (Corrigan, 2004).

Initially, asthma was classified into intrinsic and extrinsic phenotypes based on the identification of triggering factors and the presence or absence of allergic sensitization (Ricciardolo et al., 2023; Foppiano and Schaub, 2023). However, advances in the understanding of immunopathogenesis made it possible to recognize that the exacerbation of asthmatic signs and symptoms is associated with distinct immune response profiles (Bourdin et al., 2024; Fitzpatrick et al., 2002). In this context, predominantly eosinophilic and neutrophilic inflammatory patterns were described, generally linked to type 2 (Th2) and non-type 2 (Th1/Th17) immune responses, respectively (Woodruff et al., 2009). This conceptual shift supported a more robust classification of severe asthma, based not only on clinical criteria but also on the underlying inflammatory and immunological mechanisms (Bourdin et al., 2024; Fitzpatrick et al., 2002; Liu et al., 2017; Global Initiative for Asthma, 2019).

Since 2019, asthma has been classified into two main subtypes: Th2-high (allergic) asthma and Th2-low (non-allergic) asthma (Drake and Kita, 2017). Allergic asthma is associated with the production of proinflammatory cytokines such as IL-4, IL-5, and IL-13, as well as with the presence of IgE and eosinophils, the latter of which are considered biomarkers in patients with the Th2-high or allergic asthma subtype. In contrast, non-allergic or Th2-low asthma is characterized by the absence of a predisposing family history of atopic disease, a non-eosinophilic inflammatory pattern, and a Th17-mediated immune response, which has been associated with increased IL-33 levels (Fitzpatrick et al., 2002). IL-33 is a multifunctional cytokine involved in a wide range of biological processes, including the development and regulation of immune responses, the maintenance of tissue homeostasis, and tissue repair and remodeling (Liew et al., 2016). It has also been implicated in the pathogenesis of several diseases, including allergic airway diseases (Mexico City Childhood Asthma Study et al., 2011). In humans, the relevance of IL-33 in these conditions is supported by genome-wide association studies (GWAS), which have shown that IL-33 and its receptor ST2 are associated with asthma susceptibility (Préfontaine et al., 2010). IL-33 is produced by several cell types, including epithelial cells, particularly those of the respiratory tract. In response to viral infections, these cells increase IL-33 production, which has been observed mainly in the nasal and bronchial epithelium of patients with allergic asthma, allergic rhinitis, and chronic obstructive pulmonary disease (COPD) (Liew et al., 2016; Mexico City Childhood Asthma Study et al., 2011; Fan et al., 2023); also, these illnesses are characterized by airway remodeling which is a crucial pathological hallmark. Increased IL-33 levels promote Th17 responses, which in turn contribute to neutrophilic inflammation. IL-17A, produced by Th17 cells, promotes the activation of epithelial cells, fibroblasts, and neutrophils, leading to the release of neutrophil-associated chemokines such as IL-6 and CXCL8 (Liew et al., 2016; Chesné et al., 2014). This response enhances neutrophil recruitment and activation in the airways, where these cells secrete bioactive mediators, including neutrophil elastases, thereby contributing to lung tissue damage and excessive mucus production (Chesné et al., 2014; Manti and Piedimonte, 2022) (**Figure 1**).

**Figure 1:**
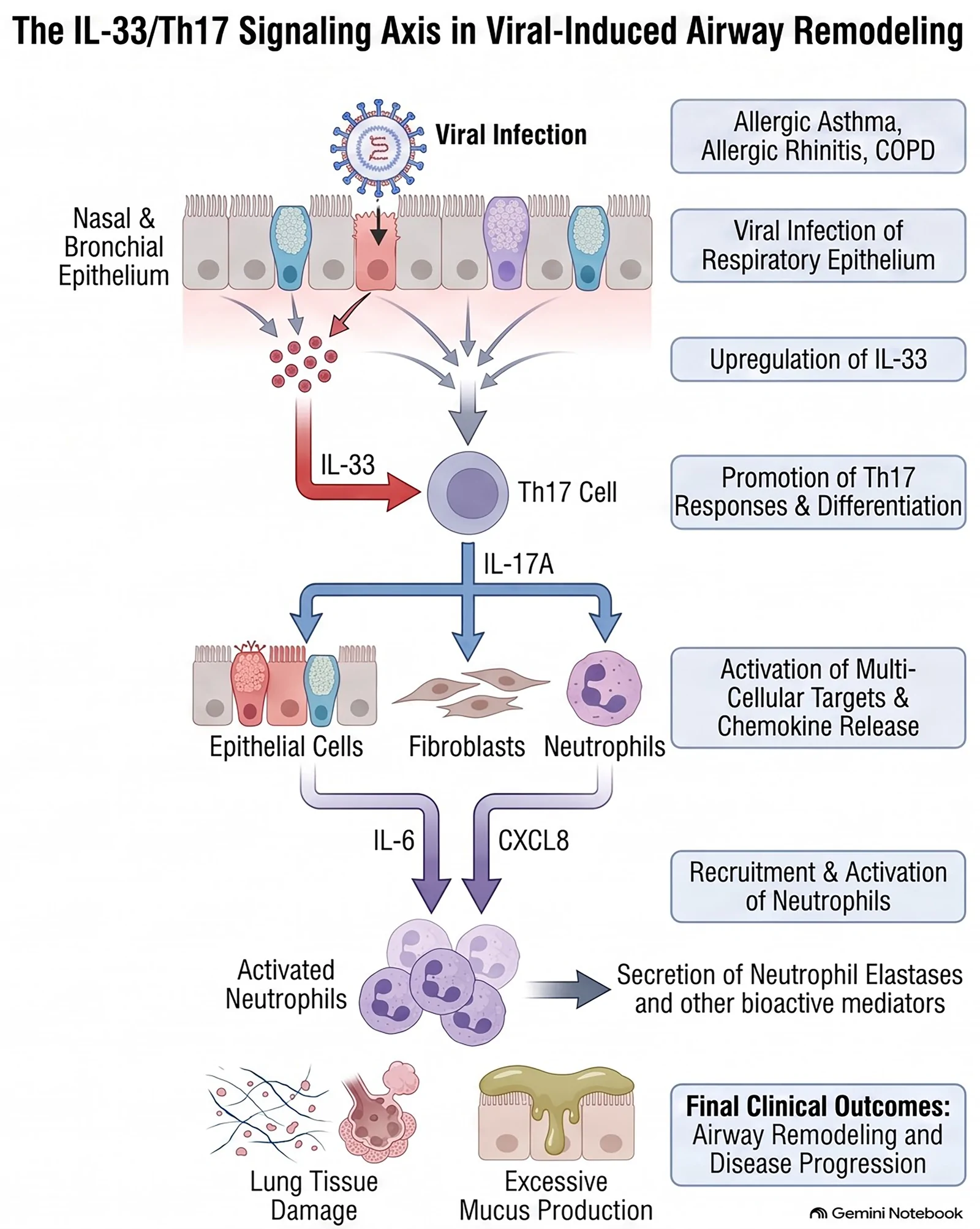
Schematic representation of the IL-33/Th17 signaling axis and airway remodeling in inflammatory respiratory diseases. In response to viral infections, respiratory epithelial cells—particularly in the nasal and bronchial epithelium—upregulate the production of interleukin-33 (IL-33). This elevated IL-33 release is a prominent feature in patients with allergic asthma, allergic rhinitis, and chronic obstructive pulmonary disease (COPD), conditions fundamentally characterized by airway remodeling. Increased IL-33 levels promote Th17 cell responses, driving IL-17A production and subsequent neutrophilic inflammation. IL-17A activates epithelial cells, fibroblasts, and neutrophils, triggering the secretion of neutrophil-associated chemokines such as IL-6 and CXCL8. This cascade enhances the recruitment and activation of neutrophils in the airways, which secrete bioactive mediators, including neutrophil elastases, ultimately leading to progressive lung tissue damage and excessive mucus production.

Lastly, RSV and HMPV may be associated not only with acute exacerbations in children with asthma, but also with the initiation of non-atopic asthma when infection occurs early in life. Early-life infection with these viruses may induce airway hyperreactivity and inflammation, thereby contributing to asthma development (Porsbjerg et al., 2022; Varricchi et al., 2022**;** Muñoz-Quiles et al., 2023). However, although their role in the onset of non-atopic asthma through other independent causal pathways has long been suspected, the association remains less well established. RSV infection affects a diversity of cells in the respiratory system. Among these are ciliated cells, which in response to viral infection produce and release proinflammatory cytokines such as TNF-alfa, IL-33 (epithelium-derived alarmin), and thymic stromal lymphopoietin (Porsbjerg et al., 2022). These cytokines promote a Th2-type inflammatory response and the recruitment of neutrophils and eosinophils (Lachowicz-Scroggins et al., 2019; Wan et al., 2020). Neutrophils are also susceptible to RSV infection and become activated upon infection, leading to NETosis and the formation of neutrophil extracellular traps (NETs). The peak of these processes coincides with the highest viral load, greater clinical severity, and lung tissue damage (Chesné et al., 2014; Lehman and Segal, 2020). Equally important, HMPV should be considered a relevant causal agent in disease initiation and/or a trigger of respiratory disease exacerbations. In addition, HMPV persistence may represent another risk factor contributing to the development of non-atopic asthma or neutrophilic asthma and lung tissue damage (Rudd et al., 2017).

## 4 Non-atopic asthma or neutrophilic asthma: lung tissue damage by NETS

During respiratory infections by bacteria, fungi or virus, the innate immune response plays a crucial role as the first line of defence. Among the different types of cells that participate in the innate immune response, neutrophils represent the first line of defence against infectious agents (Cambier et al., 2024). These cells migrate in response to the soluble chemotactic factors that are, in turn, produced by the respiratory cells in response to a viral infection. The infected epithelial cells produce CXCL8, thus the neutrophils migrate to the infected tissue, and the activated neutrophils secrete different molecules, including metalloproteinase, ROS (Reactive Oxygen Species) and CXCL8 (Qi et al., 2010; Penders et al., 2025). Moreover, not only are these cells susceptible to being infected by some respiratory viruses like RSV, they also, in consequence, produce CXCL 8 and metalloproteinase which participate in the CXCL-8 processing. The excessive presence of neutrophils, ROS and the increased levels of CXCL-8 contribute to developing chronic inflammatory airway epithelium, which is considered a risk factor in non-atopic asthma, also called neutrophilic asthma (Th2-low) (Lachowicz-Scroggins et al., 2019; Qi et al., 2010; Penders et al., 2025). Furthermore, the activated neutrophils release NETs, composed of a host double-stranded DNA scaffold, histones, and associated granule proteins such as myeloperoxidase, and elastase in a process sometimes called NETosis (Zuo et al., 2020; Dworski et al., 2011). Recently, some studies have reported the presence of NETs in bronchial biopsies of asthmatic patients (Hudock et al., 2020). The NETs induced the CXCL8 production from human airway epithelial cells (Zwaans et al., 2014), promoting the inflammatory process and the damage to the respiratory epithelium. The contribution of NETs to the inflammatory process and their detection in samples from asthmatics patients suggests that neutrophils and their products might participate mainly in the pathophysiology of Th2-low asthmatics (Zhang et al., 2026). In sum, Th2-low asthma is distinguished by an inflammatory process, as a consequence of neutrophilic presence and activation of Th1 and/or Th17 cells, ILC1s (Innate Lymphoid Cells), ILC3s and the release of specific cytokines (e.g., CXCL8, IL-17) (Lachowicz-Scroggins et al., 2019). It is also important to mention that airway neutrophilia and the induction of NETS could play a pathogenic role not only in Th2-low asthma but also in COPD patients (Lachowicz-Scroggins et al., 2019; Trivedi et al., 2021). In COPD, the accumulation and activation of neutrophils during exacerbation may amplify airway inflammation and mucus production. These processes can contribute to greater exacerbation, severity and progressive lung tissue damage (Lachowicz-Scroggins et al., 2019; Wiseman et al., 2024; Turner et al., 2024).

In conclusion, chronic airway inflammation, which may result from various factors such as respiratory virus infection, the inflammatory immune response (including cells and soluble mediators such as cytokines and chemokines), and NETosis, may contribute to tissue remodeling and disease progression, both in neutrophilic (Th2-low) asthma and in COPD (Feng et al., 2025). Regarding the role of the NETosis in these chronic respiratory diseases, it is important to point out that when NET release is dysregulated or excessive, it emerges as a pivotal driver of immunopathology in respiratory diseases. The NETosis enriched airway secretions exacerbate epithelial injury, mucus hypersecretion, and impair mucociliary clearance, thereby accelerating airway obstruction and tissue remodeling. Moreover, the excessive neutrophil infiltration and NET accumulation disrupt the alveolar capillary (Kim and Kim, 2026). Recently, the findings of Aghayan AH et al, demonstrate that NETs have specific toxic effects on MSCs, including increased apoptosis and necrosis, mitochondrial dysfunction, impaired migration capacity, and a reduction in antioxidant defenses. Exploring strategies to mitigate NET-mediated toxicity might also be valuable for refining MSC applications under inflammatory conditions (Aghayan et al., 2024).

Therefore, mesenchymal stem cells are critical to modulating the immune response and their participation in respiratory tissue regeneration, which may contribute to the regulation of lung homeostasis.

## 5 The mesenchymal stem cells might be a key participant in the recovery of the respiratory tissue homeostasis

The primary function of the lung is oxygen–carbon dioxide gas exchange. During inspiration, the lung is continuously exposed to airborne pathogens, which enter through the airways and reach the sites of gas exchange: the alveoli (Zepp and Morrisey, 2019). Protection of the lungs against respiratory pathogens is mediated by epithelial cells, resident and recruited immune cells, endothelial cells, and mesenchymal cells. In the lungs, basal cells are epithelial cells that represent resident mesenchymal stem/stromal cells (LrMSC) and, owing to their multipotent capacity, can differentiate into multiciliate cells and club cells, which produce antimicrobial, and immunomodulatory peptides and goblet cells, which produce mucus (Zepp and Morrisey, 2019; Doherty et al., 2023) (**Table 1**). In addition, type II alveolar epithelial cells produce surfactant proteins and act as facultative progenitors of alveolar epithelial cells. Also, the Lr-MSCs exert their effects through both cell-to-cell interactions and paracrine signaling, thereby regulating the activated state of the neighboring epithelial, endothelial, and immune cells resulting in cytoprotection, proliferation and repair (Sveiven and Nordgren, 2020; Doherty et al., 2023). Moreover, substantial evidence supports the critical role of Lr-MSCs residing in the lower airways as preserving pulmonary homeostasis, including lung structural integrity and tissue regeneration (Walewska et al., 2025; Ding et al., 2022). Furthermore, these cells are considered hypoimmunogenic because they produce low levels of inflammatory mediators until their immunomodulatory functions are activated by IL-1β, IFNγ, and TNFα (Ding et al., 2022; Hackel et al., 2023, Hackel et al., 2021; Zarate et al., 2022). These cytokines are produced by activated cells in response to airway pathogens and/or allergic factors.

**Table 1:** Definitions of the mesenchymal cell nomenclature used in the review.

Term	Definition
Mesenchymal stromal cell (MSC)	Non-hematopoietic, multipotent, self-renewing mesenchymal stromal cells are found in various tissues of the body. MSCs are plastic adherent and have a positive surface expression of CD73, CD90, and CD105, whilst negative for immune and endothelial cell surface markers (including CD45, CD34, CD14, or CD11b, CD79alpha or CD19, and HLA-DR), and the ability to differentiate into osteoblasts, adipocytes, and chondroblasts in vitro.
Mesenchymal stem cell	Cells meeting the criteria for mesenchymal stromal cells, in addition to demonstrable progenitor cell functionality using both in vitro and in vivo techniques.
Lung resident-MSC (LR-MSC)	Cells meeting the criteria for mesenchymal stromal cells which have been isolated specifically from lung tissue or fluid collected from lung tissue.
Lung pericyte	Multipotent perivascular cells that are embedded in the basement membrane of pulmonary and systemic capillaries and express markers including PDGFR-β and NG2+.
Mesenchymal progenitor cell (MPC)	Progenitor cells give rise to all mesenchymal lineages in the lung. These multipotent mesenchymal progenitors are characterized as Gli1+ Wnt2+ Isl1+ expressing in embryonic tissue but express various markers in the adult lung depending on location.

Taken from Phinney et al. (2023).

During acute viral respiratory infections, such as those caused by RSV and HMPV, specific cell lineages participate in immune responses and resolution, whereas resident or recruited lung mesenchymal cells contribute to tissue repair and exert immunomodulatory functions. However, mesenchymal cells are susceptible and permissive to infection by these viruses, a process which might be associated to the expression of surface molecules such as Toll-like receptors 2, 4, and 6, which, in turn, may function as viral entry molecules (Brügger et al., 2021; Lee et al., 2011; Olejnik et al., 2018). Consequently, infection triggers the production of soluble immune mediators that may have biological effects on the pulmonary microenvironment, thereby altering tissue homeostasis (Lee et al., 2011) (**Table 2**).

**Table 2:** Comparative impact of RSV and HMPV on MSC function.

Feature	RSV	HMPV
Evidence of MSC/LrMSC infection	Direct evidence in human MSCs and lung-resident MSC models; RSV can replicate in MSCs and engage pulmonary MSC populations in vivo [1–3].	Direct evidence in MSCs is emerging, but direct in vivo evidence in lung-resident MSCs remains limited [4].
Molecular pathways affected	Antiviral signaling, innate immune sensing, Toll-like receptor expression, cytoskeletal organization, and repair/remodeling-associated transcriptional programs [1–3].	Cytoskeletal architecture, surface phenotype, and growth factor-associated reparative pathways [4].
Cytokines, mediators, or growth factors altered	IL-1β, IL-6, IL-8, SDF-1, IFN-β, and IDO-dependent immunomodulatory responses [1].	Decreased EGF and VEGF; increased Ang-2 and PDGF-AA [4].
Potential effect on repair/remodeling	May alter MSC immunomodulation, repair responses, differentiation programs, angiogenesis, and remodeling-related pathways; causal role in asthma/COPD remains unproven [1–3,5].	May impair or reprogram reparative MSC properties; contribution to airway remodeling remains hypothetical and requires validation [4,5–7].
Strength of evidence	Stronger: direct cellular evidence plus in vivo lung-resident MSC experimental support.	More limited: in vitro MSC evidence; lung-resident MSC-specific role remains a future direction.

[1]. Cheung et al., 2016.

[2]. Rosales Velázquez et al., 2025.

[3]. Brügger et al., 2021.

[4]. López-Mejia et al., 2026.

[5]. Doherty et al., 2023.

[6]. Kahn, 2006.

[7]. Schuster and Williams, 2014.

This event is particularly relevant in patients with non-atopic asthma and COPD who experience recurrent respiratory reinfections, in whom viral infection of different lung cell types, particularly mesenchymal stem/stromal cells, may contribute to changes in the microenvironment and impair the ability of these cells to participate efficiently in tissue regeneration, ultimately affecting tissue homeostasis. Available *in vitro* evidence indicates that HMPV can alter MSC biology in ways that are relevant to tissue repair. HMPV infection has been reported to modify cytoskeletal architecture, affect surface phenotype, and alter the secretion of growth factors involved in reparative and remodeling processes, including decreased EGF and VEGF and increased Ang-2 and PDGF-AA, these findings suggest that HMPV may impair or reprogram MSC reparative properties (López-Mejia et al., 2026). According to some reports, chronic lung diseases such as non-atopic asthma (Th2-low) and COPD, the Lr-MSC have reduced immunomodulatory functions and diminished repair responses (Doherty et al., 2023), which may lead to disease progression. The study reported by Walewska et al., 2025, demonstrated that asthmatic inflammation reduces the number of MSCs residing in the lower airways. The implicated mechanism in this process is still unknown, but the authors have shown that the remaining MSCs exhibit a loss of functional properties, potentially contributing to the progression of asthmatic inflammation by losing their regulatory role in its resolution.

## 6 Summary

First, the strongest direct evidence currently available supports the ability of RSV to infect and functionally alter human MSCs and lung-resident MSCs. RSV has been shown to replicate in human MSCs and modify their cytokine, chemokine, antiviral, and immunomodulatory responses, including changes in IL-1β, IL-6, IL-8, SDF-1, IFN-β, and IDO-dependent pathways (Cheung et al., 2016). In addition, RSV-infected human MSCs overexpress Toll-like receptors and show altered cytoskeletal organization, supporting the concept that RSV infection modifies innate immune sensing and cellular architecture in MSCs (Rosales Velázquez et al., 2025). Furthermore, studies using pediatric lung-resident MSCs and an ovine model of human RSV infection demonstrated that RSV could invade and activate lung-resident MSCs *in vivo*, expand the pulmonary MSC pool, and induce transcriptional programs associated with antiviral defense, tissue repair, differentiation, remodeling, and angiogenesis (Brügger et al., 2021). Together, these findings provide direct cellular and experimental evidence that RSV infection can reprogram MSC biology.

Second; the evidence for HMPV is emerging but more limited. Available data indicate that HMPV infection can modify MSC cytoskeletal architecture, alter surface phenotype, and affect the secretion of growth factors involved in tissue repair, including decreased EGF and VEGF and increased Ang-2 and PDGF-AA (López-Mejia et al., 2026). These findings suggest that HMPV may impair or reprogram MSC reparative properties. However, unlike RSV, direct *in vivo* evidence connecting HMPV-infected MSCs with airway remodeling, asthma development, or COPD progression remains insufficient. Therefore, the HMPV-associated MSC dysfunction might represent an emerging hypothesis and should be explicitly identified as a research gap.

Third: Epidemiological and clinical studies have shown that severe early-life RSV bronchiolitis is associated with recurrent wheezing and later asthma (Makrinioti et al., 2022**;** Zhou et al., 2021). However, these studies do not demonstrate that MSC infection is the causal mediator of these outcomes. Similarly, studies and reviews implicating lung-resident MSC dysfunction in chronic lung disease support a possible mechanistic link between impaired repair, altered immunomodulation, profibrotic differentiation, and airway remodeling, but they do not establish that viral infection of MSCs directly causes asthma or COPD progression (Doherty et al., 2023).

Fourth: Future studies are needed, including lineage-tracing models, conditional viral infection systems, lung organoids, epithelial–MSC co-culture models, single-cell longitudinal analyses, and prospective human studies linking viral infection, MSC dysfunction, remodeling biomarkers, and long-term obstructive airway outcomes.

## 7 Conclusion

Human respiratory viruses, particularly Respiratory Syncytial Virus and human Metapneumovirus (HMPV), are not only triggers of exacerbations in atopic asthma (Th2-high),but may also contribute to the initiation and progression of non-atopic or neutrophilic asthma (Th2-low) and COPD. During acute and recurrent viral infections, the lower airways produce proinflammatory cytokines, including IL-1, TNF-α, IL-33, and IL-17A, as well as chemokines such as CXCL1 and CXCL8. Because CXCL8 promotes neutrophil recruitment, neutrophilic inflammation represents a key feature of non-atopic asthma. This inflammatory microenvironment, together with soluble mediators and activated immune cells, disrupts lung homeostasis and creates a context in which mesenchymal stem/stromal cells become essential for tissue repair, regeneration, and immunomodulation. However, these protective properties may be reduced during chronic inflammation, as reported in non-atopic asthma and COPD (Doherty et al., 2023). In addition, MSCs are susceptible and permissive to RSV and HMPV infection, suggesting that viral infection may further impair their reparative and immunomodulatory functions. Therefore, the combined effects of chronic inflammation and viral infection of MSCs may contribute to persistent airway remodeling, impaired restoration of lung homeostasis, and progression of chronic respiratory disease **Figure 2**.

**Figure 2:**
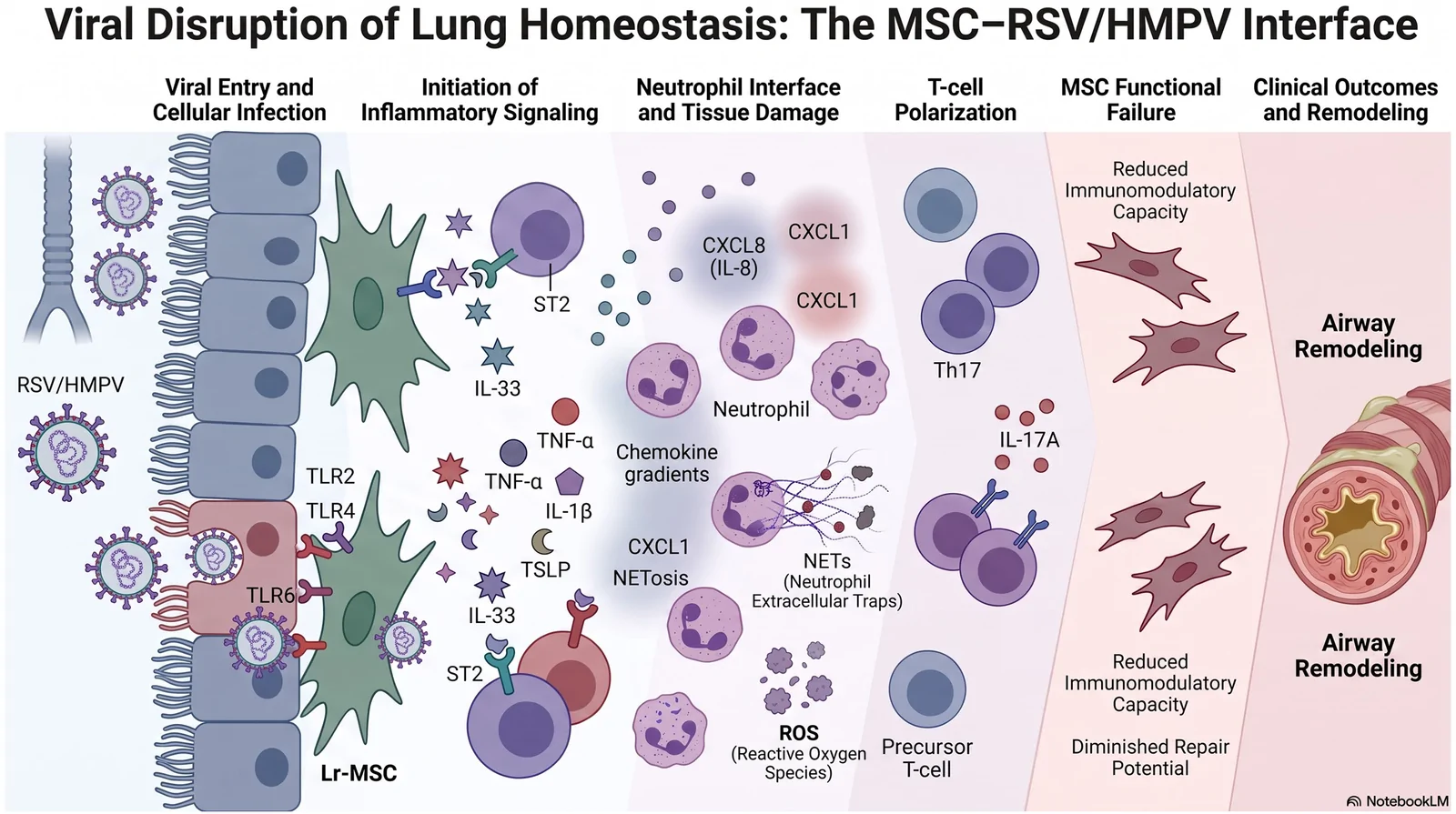
Viral disruption of MSC-mediated lung homeostasis and its role in chronic respiratory diseases. The schematic illustrates the pathophysiological axis triggered by Respiratory Syncytial Virus (RSV) and human Metapneumovirus (HMPV) infections in the lung microenvironment. (1) Viral Entry and Permissiveness: RSV and HMPV particles infect both ciliated epithelial cells and lung-resident Mesenchymal Stem Cells (Lr-MSCs). On Lr-MSCs, the viruses utilize Toll-like receptors 2, 4, and 6 (TLR2, TLR4, TLR6) as entry molecules, facilitating direct infection of these progenitor cells. (2) Proinflammatory Cascade: Infected epithelial cells release potent alarmins and cytokines, including IL-33, TSLP, TNF-α, and IL-1β. IL-33 interacts with its receptor, ST2, promoting susceptibility to airway disease. (3) Neutrophilic Inflammation: The secretion of chemokines CXCL1 and CXCL8 (IL-8) drives the recruitment and activation of neutrophils. Activated neutrophils undergo NETosis, releasing Neutrophil Extracellular Traps (NETs) and Reactive Oxygen Species (ROS), which exacerbate epithelial barrier damage and promote a chronic inflammatory state. (4) Adaptive Immune Polarization: The inflammatory milieu favors a Th17-mediated response, characterized by high levels of IL-17A, which further amplifies neutrophilic recruitment and tissue injury. (5) Lr-MSC Dysfunction: Direct viral infection and exposure to the chronic inflammatory microenvironment lead to a loss of plasticity, reduced immunomodulatory capacity, and diminished repair responses in Lr-MSCs. (6) Clinical Outcomes: The failure of Lr-MSC-mediated homeostasis and persistent inflammation culminate in extensive Airway Remodeling, driving the progression of Chronic Obstructive Pulmonary Disease (COPD) and the development of Th2-low (non-atopic) asthma.
